# Microbiota as Drivers and as Therapeutic Targets in Ocular and Tissue Specific Autoimmunity

**DOI:** 10.3389/fcell.2020.606751

**Published:** 2021-02-05

**Authors:** Ryan Salvador, Amy Zhang, Reiko Horai, Rachel R. Caspi

**Affiliations:** Laboratory of Immunology, National Eye Institute, National Institutes of Health, Bethesda, MD, United States

**Keywords:** microbiota, autoimmune uveitis, tissue-specific autoimmunity, animal models, multi-omic approach, treatment intervention

## Abstract

Autoimmune uveitis is a major cause of blindness in humans. Activation of retina-specific autoreactive T cells by commensal microbiota has been shown to trigger uveitis in mice. Although a culprit microbe and/or its immunogenic antigen remains to be identified, studies from inducible and spontaneous mouse models suggest the potential of microbiota-modulating therapies for treating ocular autoimmune disease. In this review, we summarize recent findings on the contribution of microbiota to T cell-driven, tissue-specific autoimmunity, with an emphasis on autoimmune uveitis, and analyze microbiota-altering interventions, including antibiotics, probiotics, and microbiota-derived metabolites (e.g., short-chain fatty acids), which have been shown to be effective in other autoimmune diseases. We also discuss the need to explore more translational animal models as well as to integrate various datasets (microbiomic, transcriptomic, proteomic, metabolomic, and other cellular measurements) to gain a better understanding of how microbiota can directly or indirectly modulate the immune system and contribute to the onset of disease. It is hoped that deeper understanding of these interactions may lead to more effective treatment interventions.

## Introduction

Development of autoimmune diseases is influenced by both genetic and environmental factors (Kamada et al., 2013). In genetically predisposed hosts, autoimmunity is more likely triggered under the influence of environmental components, such as diet, commensal microbiota, infection or injury, and/or exposure to chemicals and medications (Lin et al., 2014; Rosenbaum et al., 2016). These elements together play an important role in the development of disease, but the etiology largely remains uncertain.

Commensal microbiota colonize the barrier surfaces of our body that come into contact with the environment (Sprouse et al., 2019). The most densely populated tissue is the gut, with microorganism numbers on the order of 100 trillions. A growing number of studies in the past decade implicate the dependence on gut microbiota for the development of autoimmune diseases. While effects of gut microbiota in intestinal inflammation is easily conceptualized, it becomes less straightforward when the sites of the inflammation/pathology are distant from the gut, as in tissue-specific autoimmune diseases such as uveitis or multiple sclerosis (Horai and Caspi, 2019; Sprouse et al., 2019).

The eye and brain are immunologically privileged sites. The target antigens are sequestered behind the blood-organ barrier and are not available outside the tissue to prime self-reactive T cells that have escaped from thymic negative selection and are present in circulation (Horai and Caspi, 2019). The fundamental question of where and how autoreactive T cells recognize such antigens to first become activated to initiate autoimmune reactions prompted us to study whether microbiota and/or their metabolites could serve as mimic antigens (Horai and Caspi, 2019).

T cells play a major role in the pathogenesis of autoimmunity. Th1 and Th17 cells have been intensively studied and shown to have effector and pathogenic roles (Damsker et al., 2010), whereas regulatory T cells (Treg) have immunomodulatory or inhibitory roles (Sharma and Rudra, 2018). Some commensals have been reported to preferentially induce Th17 cells or Treg cells. For example, segmented filamentous bacteria (SFB) induce Th17 cells specifically in lamina propria of the small intestine and contribute to induction of autoimmunity (Ivanov et al., 2009; Wu et al., 2010). On the other hand, certain Clostridium and Bacteroides species induce Treg and help maintain tolerance (Nutsch and Hsieh, 2012). The balance between intestinal Treg and Th17 cells is important for host-microbiota homeostasis, and lack of functional Treg in the intestine can exacerbate intestinal Th17 responses and lead to profound dysbiosis (Omenetti and Pizarro, 2015; Neumann et al., 2019). Therefore, identification of commensals that induce or modulate autoimmune responses has become an attractive approach in the immunology field.

The need for mechanistic understanding of microbiota-host interactions stimulated studies in which the identification of culprit microbes has yielded associations between commensals and effects on host immunity (Kamada et al., 2013; Horai and Caspi, 2019; Sprouse et al., 2019). However, it is necessary to synthesize and integrate data from various sources, including the microbiome, immune cell profiles and serum measurements. The network analyses and applied systems biology approaches are becoming paramount for making associations. The insights gained from integrating multiple datasets to identify microbes that are associated with autoimmune disease development may contribute to mechanistic understanding of microbe-host interactions. In this review, we discuss future approaches to integrate this information into our current knowledge, gleaned from animal models and clinical studies of autoimmune diseases, with an emphasis on uveitis.

## Microbiota as Triggers and Modulators of Tissue-Specific Autoimmunity

**FIGURE 1:**
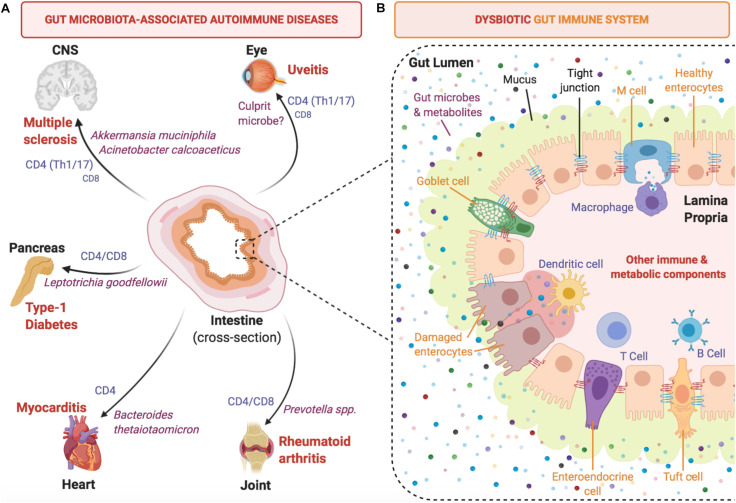
Development of autoimmune diseases is linked to gut microbiota and intestinal immunity. **(A)** Selected tissue-specific autoimmune diseases (extra-intestinal) are depicted with reported culprit microbes and effector T cell types involved in pathogenesis. **(B)** A close-up intestinal cross-section of a segment of the mammalian colon that focuses on the host-microbiota interface and highlights key immune cell mediators that contribute to the induction of pro-inflammatory cytokines under dysbiotic conditions.

Involvement of the microbiome in the development of tissue-specific autoimmune diseases has sparked interest into understanding the crosstalk between microbiota and host immunity, and how this influences overall health (Kamada et al., 2013; Horai and Caspi, 2019; Sprouse et al., 2019). During the early rise in popularity of microbiome research, numerous studies associated the presence of specific microorganisms to certain autoimmune diseases. These microorganisms are thought to contribute to autoimmune disease in various ways, including gut dysbiosis, which perturbs local gut homeostasis and may facilitate translocation of bacteria into tissues, where they fuel chronic inflammation. In addition, they may trigger autoimmunity by providing antigenic stimuli, or may modulate the influence of other triggers through their metabolites or by stimulating regulatory immune elements (summarized in Figure 1). The following examples of autoimmune disease models summarize the evidence for different levels at which microbiota may affect manifestations of disease.

### Ocular Autoimmunity

Uveitis is a general term referring to ocular inflammatory disorders of the uveal tract and one of the leading causes of the vision loss (Caspi, 2010). Autoimmune (non-infectious) uveitis is characterized by inflammation affecting the uvea (iris, ciliary body, choroid) and neuroretina. Disease is often associated with HLA haplotypes (Caspi, 2010; Lin et al., 2014; Rosenbaum et al., 2016). Clinical symptoms include inflammation of uvea and retina, vasculitis, choroiditis, resulting in poor visual prognosis (Caspi, 2010). It can be part of a systemic autoimmune syndrome, such as Behcet’s disease and Vogt-Koyanagi-Harada disease, or can affect only the eye, such as birdshot retinochroidopathy and sympathetic ophthalmia (Caspi, 2010). Contribution of microbiota to the development of autoimmune uveitis in animal models and humans has been intensively discussed in a recent review (Horai and Caspi, 2019). Here we summarize our findings implicating microbial stimuli in activating autoreactive T cells in uveitis (Horai et al., 2015), which have been followed by reports that connected specific commensals as stimuli in other tissue-specific autoimmune diseases.

Among the experimental mouse strains tested, B10.RIII is the most highly susceptible to experimental autoimmune uveitis (EAU) induced by active immunization with the retinal antigen, interphotoreceptor retinoid binding protein (IRBP), without need for additional stimulation with pertussis toxin (Horai and Caspi, 2010). Our laboratory developed a T cell receptor (TCR) transgenic mouse line, R161H, that expresses a TCR specific for IRBP peptide on the B10.RIII background, where all mice develop spontaneous uveitis by 2 months of age (Horai et al., 2013). Due to its spontaneous nature, the R161H model is particularly useful to study natural triggers that can contribute to uveitis, and has been instrumental in implicating the commensal microbiome as a trigger of uveitis (Horai et al., 2015). Treatment with oral antibiotics or rearing under germ-free (GF) conditions resulted in lower severity of disease and fewer Th17 cells in the intestinal lamina propria. Importantly, retina-specific T cells appeared to receive a signal through their clonotypic TCR in the intestine, and this was not dependent on expression of autologous IRBP (Horai et al., 2013, 2015). This constitutes evidence that microbial components may play a role as mimic antigen(s) to activate retina-specific T cells passing through the gut, which then migrate to the eye and trigger uveitis.

Microbiota may also modulate uveitis in ways other than providing a source of surrogate antigen. In immunization-induced EAU, where disease is triggered by antigen administered peripherally, oral antibiotic treatment (ABX) has the potential to inhibit disease. However, different groups reported conflicting results: long-term ABX in wild-type B10.RIII mice did not affect EAU development following IRBP immunization (Horai et al., 2015), but short-term ABX appeared to inhibit EAU development (Nakamura et al., 2016). Whether these findings can be attributed to confounding experimental factors, including the environment in the respective animal facilities (microbial flora, diet etc.), remains to be determined. In another study with an induced model of EAU, morphological and inflammatory changes were detected in the gut upon immunization in the presence of adjuvant (microbial component), suggesting leakiness of the gut associated with dysbiosis (Janowitz et al., 2019). Kaede transgenic mice, which express a photoconvertible fluorescent reporter protein “Kaede” that allows for the local photoconversion and subsequent tracking of photoconverted cells in other locations, were used to demonstrate that there is an unexpectedly broad movement of leukocytes to and from the gut to many peripheral sites under homeostatic conditions (Morton et al., 2014). Kaede mice were used to demonstrate that there is enhanced leukocyte trafficking from the intestine to the eye during EAU (Nakamura et al., 2017). Although there were confounding issues in this experimental system, as discussed elsewhere (Horai and Caspi, 2019), in the aggregate, the experiments described above lead to the conclusion that gut microbiota can modulate the responses and behavior of uveitogenic T cells at several levels by providing adaptive and innate stimuli, as well as possible regulatory effects.

### Non-Ocular Tissue Specific Autoimmune Disease Models

Pathogenesis of EAU shares a lot of similarity with experimental autoimmune encephalomyelitis (EAE), a model for multiple sclerosis (MS), a disease affecting the brain and spinal cord which are also considered immune privileged tissues (Glenn and Mowry, 2016). The eye, like the brain, is part of the central nervous system (CNS). Similar to EAU, EAE is a T cell mediated autoimmune disease model, and can be induced by active immunization with CNS antigens. In both models, activated tissue-specific effector T cells that can recognize self-antigens within the CNS pass through a blood-organ barrier and attack the target tissue to cause inflammation (Caspi, 2010; Glenn and Mowry, 2016). Using antibiotic treatment, Berer et al. (2011) demonstrated in a spontaneous model of EAE in myelin basic protein (MBP) TCR transgenic mice, that disease is dependent on presence of intestinal microbiota. They postulated antigenic mimicry, but did not present direct evidence to support this. Furthermore, antibody production to MBP required expression of endogenous myelin in addition to microbiota, suggesting that, in contrast to R161H uveitis, the inciting antigen could be autologous, and the microbiota might be playing an auxiliary or “adjuvant” role. In the induced (active) and adoptive transfer (passive) EAE models, there was evidence for increased intestinal permeability (Nouri et al., 2014), suggesting that bacterial translocation might occur and possibly contribute to disease. Whatever the stimuli, however, it appears that human gut microbiota were able to provide them. Human gut flora inoculated into GF MBP TCR transgenic mice was able to support spontaneous EAE development (Berer et al., 2017; Cekanaviciute et al., 2017) and microbial candidates (*Akkermansia muciniphila* and *Acinetobacter calcoaceticus*) that were enriched in MS patients induced proinflammatory responses in human peripheral blood mononuclear cells and in mono-colonized mice (Cekanaviciute et al., 2017).

While in EAE evidence for microbial mimicry by commensals is questionable, and in EAU we were unable to identify the bacteria that contained the mimic of the pathogenic IRBP peptide, microbial mimics were identified in other autoimmune disease models. Examples are autoimmune myocarditis and type 1 diabetes (T1D) in non-obese diabetic (NOD) mice. A transgenic mouse model that expresses a TCR specific for myosin heavy chain 6 (MYH6) was reported to develop spontaneous cardiomyopathy. *Bacteroides thetaiotaomicron*, a commensal shown to have been enriched in myocarditis patients, expressed a β-galactosidase peptide that mimics the host protein MYH6 and activates antigen-specific T cells that enhance disease (Gil-Cruz et al., 2019). In NOD mice, a magnesium transporter peptide from *Fusobacteria* was identified as a mimic of islet-specific glucose-6-phosphatase catalytic subunit-related protein (IGRP), a target antigen expressed in pancreatic beta cells. NOD mice expressing a TCR specific to IGRP in their CD8^+^ T cells developed highly accelerated diabetes when associated with gut microbiota enriched with *Fusobacteria* (Tai et al., 2016). Nevertheless, molecular mimicry does not exclude contribution of other mechanisms to T1D. In a streptozotocin-induced T1D model, translocation of bacteria to peripheral lymph nodes was observed due to altered microbiota and compromised gut integrity. Antibiotic treatment was protective, and this was reversed by re-administration of a microbial ligand, muramyl dipeptide (Costa et al., 2016). Another mechanism that modulates T1D involves the effects of sex hormones on microbiota. T1D in mice characteristically show a pronounced gender bias, affecting mainly females. Reports from several groups have led to the conclusion that androgens shape the microbial repertoire in males toward a composition that affords protection, which occurs in part through TLR-dependent mechanisms (Markle et al., 2013; Yurkovetskiy et al., 2013; Burrows et al., 2015).

Reports showing associations between specific gut commensals and human autoimmune diseases continue to multiply. A detailed enumeration of the particular microbes that are associated with diverse diseases is beyond the scope of this article, and the subject has recently been reviewed (Zhang et al., 2020). It should be kept in mind, however, that causal relationships cannot be firmly established simply by studying the human cohorts, and mechanistic explorations often need to resort to animal experiments. Transplantation of human flora into animal models of disease provide an opportunity to study cause-and-effect relationships.

## Establishing Causative Effects Between Gut Microbes and Autoimmune Diseases

It is very difficult to rigorously establish cause-and-effect relationships in human studies. Published studies on the role of microbiota in human uveitis fail to establish causality. Nevertheless, many reports are suggestive, and hypotheses can be supported by well controlled experiments in animal models.

### Germ-Free and Antibiotics Approaches

Early studies linking gut microbiota to autoimmune diseases were performed by direct perturbation of the microbiota in different experimental models. If there is a positive correlation between the microbiota and progression of disease, ABX or GF rearing is expected to abrogate disease progression (Horai et al., 2015; Seifert et al., 2018). However, there are limitations to both approaches. A concern of using GF mice is that normal development of the immune system is strongly dependent on microbiome, such that responses of GF mice to immune stimuli in adulthood can be blunted (Dzidic et al., 2018; Levan et al., 2019).

ABX can also have unintended effects on host immune responses. As discussed above, duration of ABX can result in different disease outcomes in the EAU model. A separate study investigating the effects of ABX on host immune status in mice showed that body weight and spleen size were decreased, while cecum (among other intestinal sections) was enlarged within 1 week of ABX. However, after the 1st week, continued ABX did not seem to worsen gut inflammation, and colon enterocyte transcriptome was restored to a state closer to non-treated controls (Tao et al., 2020). Thus, it must be taken into consideration that microbiota-depletion experiments by ABX can have effects on immune phenotypes that may, or may not, be temporary.

Although, unlike GF mice, ABX animals have the advantage of a fully developed immune system, ABX does not eliminate fungal or viral species. Therefore, ABX models cannot rule out the immune interactions between the host and commensal fungi/viruses, confounding correlation studies.

### Humanized Gnotobiotic Models

In hopes of extrapolating results from animal models to human subjects, a “humanized” approach is often applied, in which human microbiota is established in GF or ABX animals (Turnbaugh et al., 2009; Walter et al., 2020). A typical humanized gnotobiotic study involves reconstitution of GF rodents with fecal matter from either healthy donors or patients, followed by comparisons of transferred microbiota, established microbiota and disease readouts in the animal recipients. Such gnotobiotic human microbiota-associated (HMA) mice have been widely used in the past decade to study causality and mechanisms of (gut) microbiome-disease associations (Walter et al., 2020). One of the initial studies exemplifying the use of HMA mice centered on obesity and diet. HMA mice reproduced much of the human donor’s fecal bacterial diversity and such colonization is heritable through lab mouse generations. The study also showed that mice colonized with humanized microbiota from Western diet-fed donors recapitulated the trait of adiposity observed in the donors (Turnbaugh et al., 2009).

Besides metabolic syndrome to which diet is often the key contributing factor, gut microbiome has been shown to influence a range of autoimmune diseases in sites distant from the gut, such as rheumatoid arthritis (Wu et al., 2010), autoimmune uveitis (Horai et al., 2015), MS (Berer et al., 2017), and lupus (Greiling et al., 2018). For example, the MS study involving HMA showed that GF relapsing-remitting (RR) SJL/J MBP TCR transgenic mice reconstituted with fecal samples from MS patients had higher incidence of spontaneous EAE than healthy stool-recipient gnotobiotic mice (Berer et al., 2017). In autoimmune uveitis, fecal samples from active patients were also shown to exacerbate EAU when transplanted into antibiotics-treated B10.RIII mice prior to immunization (Ye et al., 2018, 2020).

Ongoing or future efforts of HMA studies are to 1) mono-colonize GF animals with candidate microbes identified from health-disease comparisons to explore the potential contribution of individual microbes to the development of pathologies; and 2) evaluate whether certain microbes could serve as novel therapeutics to treat diseases, e.g., suppression of arthritis or EAE in humanized mouse models when treated with isolated human gut commensal *Prevotella histicola* (Marietta et al., 2016; Shahi et al., 2019).

### Wild Flora Reconstituted Models

Though the HMA model remains a powerful tool to study disease-microbiome associations, it may have limitations in translatability. Gnotobiotic mice harboring microbiota from heterologous species (human, rat, etc.) revealed an immature immune system, which failed to serve as a counterpart to human immunity (Chung et al., 2012). Differences between humanized mouse flora and the original donor fecal bacteria were commonly observed (Seedorf et al., 2014; Zhang et al., 2017; Lundberg et al., 2020). On the other hand, metabolomic features observed in donor fecal samples were nevertheless reproduced in corresponding gnotobiotic mice (Marcobal et al., 2013). Thus, heterologous microbiota are not equivalent to co-evolved microbiota in supporting development of immune function.

To take this concept even further, some studies indicate that laboratory SPF mouse flora is also not equivalent to the natural co-evolved mouse flora. SPF mice typically contain fewer memory cells than their wild-caught counterparts and in that regard their adult immune systems are more reminiscent of neonatal humans (Hunig, 2012). This may be the reason why outcomes of clinical trials may not recapitulate results obtained in preclinical models. A famous example is a Phase 1 clinical trial, in which CD28 superagonist treatment induced life-threatening cytokine release syndrome in human subjects, instead of the regulatory T cell activation observed in experimental animals (Hunig, 2016).

Not surprisingly, the concept of an antigen-experienced, “dirty” mouse model, long known to be more robust immunologically than SPF mice, has been receiving growing attention as a better mimic of the diverse history of infections typical of humans (Beura et al., 2016; Masopust et al., 2017). Recently, a preclinical model integrating lab mouse and its wild analog was developed, in which laboratory mouse embryos were implanted into female mice captured in the wild. The offspring born to these wild dams, dubbed “wildlings”, inherited the wild mouse flora populating the various niches (gut, skin, etc.), which was preserved through successive generations (Rosshart et al., 2019). These wildling mice not only harbored more diverse communities of bacteria, fungi and viruses compared to SPF lab mice, but they also turned out to be a better model of human immune responses, and were able to replicate a comparable outcome to the CD28 superagonist clinical trial mentioned above (Rosshart et al., 2019). Similar observations were reported by others, who co-housed lab mice with pet store mice (Huggins et al., 2019; Fiege et al., 2020), or “rewilding”, i.e., introducing lab mice into a more natural “outdoor” environment (Lin et al., 2020; Yeung et al., 2020). Results from these studies showed that “dirty” or “rewilded” mice had remarkable changes in their immune systems under different preclinical conditions, in part due to changes in the associated microbiota.

## Contribution of Mycobiome and Virome to Host Immunity

Since microbiome-disease association studies typically involved antibiotics treatment, and because of the simplicity and ease of bacteria-specific 16S marker gene survey to characterize the microbiome, attention has mostly been focused on the bacterial components within the microbial consortia. However, host-associated microbiota encompass all domains of life including bacteria, archaea, fungi, viruses and protozoa. In particular, the involvement of fungal and viral species in autoimmunity has been increasingly appreciated, strengthening the long-standing observation that fungi and viruses have the potential to trigger autoimmune diseases (Romani, 2008; Smatti et al., 2019).

A recent study presented experimental evidence that human antifungal Th17 cells cross-react with *Candida albicans*, a well-characterized fungal pathobiont in the human gut (Bacher et al., 2019). These cross-reactive T cells were shown to expand in intestinal inflammation, and responded to non-intestinal fungal species such as *Aspergillus fumigatus*, a driver of acute airway allergies, indicating the role of *C. albicans* in promoting non-intestinal immune pathologies via cross-reactivity (Bacher et al., 2019). Another study surveyed the gut mycobiome of children positive for diabetes-associated autoantibodies, and reported gut fungal dysbiosis among the group of children who progressed to clinical T1D (Honkanen et al., 2020). Specifically, *Saccharomyces* and *Candida* were found to be in high abundance, suggesting potential impact of these fungi on the development of intestinal inflammation and T1D progression (Honkanen et al., 2020).

The contribution of commensal viruses, many of them bacteriophages and dependent on the bacterial population, to host immunity is also being increasingly recognized. In healthy adults, longitudinal metagenomic analysis of fecal virome revealed its individual specificity and temporal stability (Shkoporov et al., 2019). Similar to mycobiome-focused studies, the changing gut virome has been associated with a number of autoimmune conditions, such as T1D (Kim et al., 2019) and celiac disease (Lindfors et al., 2019, 2020). The gut commensal viruses have also been shown to contribute to the modulation of immune cells in the gut, including intraepithelial lymphocytes (Liu et al., 2019).

As commonly used broad-spectrum antibiotics target and deplete specific groups of microbes from the intestine, there is usually an outgrowth of fungi in niches previously occupied by bacteria. It was reported in some studies that upon cessation of antibiotics treatment, fungal community generally declined back to their original abundance, with the exception of *Candida*, which persisted in a higher level than pre-treatment (Dollive et al., 2013). Since members of *Candida* species were shown to be major inducers of human Th17 responses, it is necessary to consider the impact of fungal expansion on intestinal and systemic immunity following antibiotics treatment. Somewhat surprisingly, after antibiotic treatment, the human gut virome seemed to undergo a minor or transient disturbance of its diversity (Abeles et al., 2015; Shkoporov et al., 2019). However, there was an observation of antibiotic resistant gene expansion from the gut virome post-antibiotics treatment, suggesting that commensal virome may play a role in acquisition of bacterial resistance to antibiotics (Abeles et al., 2015).

## Microbial Therapeutics

Microbial therapeutics are designed to correct dysbiotic flora and its effects. They can involve administration of probiotics (beneficial bacteria), prebiotics (foods that increase the beneficial flora or its products, e.g., dietary fiber) or in more extreme cases fecal microbial transplant (FMT), which has become popularized by its high degree of success in curing *Clostridium difficile* dysbiosis (Hui et al., 2019). The use of probiotics has become a popular dietary intervention with their transient and non-invasive effects, which help maintain a healthy immune system by improving gut health. Probiotics are thought to modulate immune responses, protect against physiological stress, suppress invasion of pathogens, modulate microbiota, and improve barrier function of the gut epithelium (Suez et al., 2019). Yet, how probiotics modulate the intestinal microbial balance is poorly understood, the molecular mechanisms are difficult to study, and recently their use has been questioned (Suez et al., 2018). There is a need to improve the quality of evidence, transparency, public awareness, and regulation of probiotic use (Suez et al., 2019). Although efficacy has been shown in some cases, batch to batch variation is still a concern, and wide-range applicability and effectiveness need to be determined *post hoc* by meta-analyses, as summarized in an up-to-date systematic review on ulcerative colitis cases (Iheozor-Ejiofor et al., 2020).

Nevertheless, a probiotic mixture IRT-5 (consisting of *Lactobacillus casei*, *Lactobacillus acidophilus*, *Lactobacillus reuteri*, *Bifidobacterium bifidum*, and *Streptococcus thermophilus*) was examined for ability to treat immune-mediated eye disease models. It showed therapeutic effects on autoimmune uveitis and dry eye, but had no effect on corneal graft survival. To facilitate engraftment of the probiotics, investigators pretreated mice with antibiotics for 5 days (Kim et al., 2017). Pretreatment with antibiotics was shown to be more effective than use of bowel cleansing solution or no pretreatment at all (Ji et al., 2017), but whether this treatment regimen is needed may depend on the disease model.

Bioactive microbial metabolites, such as short-chain fatty acids (SCFAs), serve as fingerprints of microbial function, and can act systemically on the host via absorption into the circulation and tissues (Koh and Backhed, 2020). SCFAs can regulate immune cell function and are associated with attenuation of inflammatory and autoimmune diseases (Correa-Oliveira et al., 2016). The SCFA propionate, also known as propionic acid (PA), administered in drinking water 3 weeks prior to immunization, was shown to attenuate EAU in mice in a strain-dependent manner, and was associated with Treg induction in the intestinal lamina propria and eye-draining lymph nodes (Nakamura et al., 2017). Propionate was only effective in C57BL/6 mice, but not in B10.RIII mice (the strain most highly susceptible to EAU). It is unclear why other SCFAs, acetate and butyrate, which could also induce Tregs and enhance intestinal barrier functions, had no effect on EAU (Nakamura et al., 2017). Translational relevance of SCFAs for neuroimmune CNS disease is supported by a study in EAE, where SCFAs, in particular PA and, to a lesser extent, i-butyric acid, were increased in the feces of CD44 knockout mice, which are resistant to disease. Resistance could be transferred to WT mice by FMT, supporting a causal relationship (Chitrala et al., 2017). In MS patients, PA levels in serum and stool were reduced compared to healthy controls (Duscha et al., 2020). Importantly, PA administration to therapy-naïve MS patients increased Treg and decreased Th1 and Th17 cells, suggesting that PA can be used as an immunomodulatory supplement for MS therapies (Duscha et al., 2020). Thus, SCFAs may act as metabolites processed by microbiota that can influence systemic immune responses (Dalile et al., 2019).

Besides probiotics and microbial metabolites, phage therapy has also come under the spotlight in an effort to combat the rising antibiotic resistance of bacteria (Kortright et al., 2019). In addition to treating refractory infections, phage therapy has been proposed in autoimmune and inflammatory disease setting, either to modulate disease-associated bacterial communities or to deliver engineered therapeutic components to treat EAE or murine collagen-induced arthritis models, respectively (Rakover et al., 2010; Miedzybrodzki et al., 2017). Due to their lack of tropism to mammalian cells, phage is considered a promising treatment and its application will be explored in a variety of disease conditions.

## An Outlook for Integration of the “-Omics” Approach

The understanding of “-omics” aims to make sense of the collective characterization and quantification of biological components that translate into the structure, function, and dynamics of an organism or organisms (Lorenzon et al., 2018). Large datasets resulting from individual experimental analyses are cross-referenced against curated databases to gain meaningful insights. Thus, “big data” approaches are at the intersection of traditional research and computational analyses. It can be envisioned that traditional research approaches combined with powerful data analysis can serve to bridge the knowledge gap and may lead to further validation studies.

### *In silico* Approach to Study Microbial-Disease Associations

The exploration of antigenic components from host-associated microbiota that potentially trigger autoimmunity can be facilitated by the cost-effective *in silico* approaches, if the T and/orB cell autoepitopes are well characterized (Gil-Cruz et al., 2019; Ruff et al., 2019). Typically, a BLAST search of the autoepitope sequence against microbial protein databases is performed, and the returned matches (usually of moderate identity scores) are examined, e.g., using the Immune Epitope Database (IEDB) to predict MHC-peptide binding affinity^1^. Putative immunogenic microbial candidates are then sorted out for further experimental interrogation. In addition to the physico-chemical properties of amino acids that often factor into linear alignment, secondary and tertiary structures of the conformational epitopes are often predicted to evaluate mimic peptide(s) and T/B cell receptor or MHC binding (Wang et al., 2011; Ruff et al., 2019).

In studies without a target autoimmune condition, homology match of microbial components is usually performed against the complete human proteome. The antigenic potential of candidate microbial peptides is then assessed based on HLA-peptide binding databases (Negi et al., 2017). Furthermore, small signaling molecules and metabolites are also found to have the potential of being convergent between microbial and human sources, and there have been ongoing efforts to mine the human metagenomes for these candidate small molecules (Cohen et al., 2017). These *in silico* approaches could not replace bench studies that could verify the immune interactions between microbial elements and host receptors, but they often shed light on the evolutionary relationship of these molecules between different host species. The abundance of bioinformatically identified homologous molecules implies a potentially underappreciated diversity of antigenic activity existing in the human microbiome consortia.

### Omics Approaches in Microbiome Studies

#### Metagenomics

Culture-independent, 16S rRNA-based characterization has dominated the field of microbiome research for the past few decades since the method came into maturity (Kuczynski et al., 2011). However, even though this technique is often competitive due to its low cost and the existence of well-curated databases, the 16S rDNA is relatively conserved, and microbial taxonomic identity constructed on 16S phylogeny is generally a mere proxy of the genus diversity present in a sample. Moreover, 16S sequencing only reveals bacteria and archaea in which 16S genes exist, and functional components of the microbial community can only be inferred from marker sequences with reference-based bioinformatic programs such as PICRUSt (Langille et al., 2013).

To achieve a fine resolution of microbial identification to species- and strain-levels, as well as to capture the complete picture of bacterial, archaeal, fungal, viral, parasitic diversity and their entire functional profiles, the shotgun metagenomics approach was developed and rapidly gained popularity during the past decade (Quince et al., 2017). Unlike the marker gene methods, shotgun metagenomics generate sequences from the pool of fragmented DNA from all the genomes in a sample, enabling reconstruction and a thorough examination of the microbial composition and the functional repertoire. These metagenomes could be further pooled together for a comprehensive analysis, to generate a blueprint of a defined niche of the host-associated microbiota (Almeida et al., 2019). Due to the high volume of its data output, shotgun metagenomic sequencing is not yet cost-effective and the downstream analyses are also computationally expensive, so that it has thus far been mostly applied to human or HMA samples. For instance, in uveitis research, metagenomic profiles were characterized in Behcet’s disease (Ye et al., 2018) and Vogt-Koyanagi-Harada disease patients (Ye et al., 2020).

#### Metatranscriptomics

If shotgun metagenomics inform researchers of who the microbes are and what they are capable of doing, shotgun metatranscriptomics is able to answer the question of what the microbes are doing “right then” in an ecosystem (Franzosa et al., 2014). Because metatranscriptomics quantify the gene expression profile of a microbiome sample, it can provide the valuable information on the sets of actively transcribed genes under different conditions from an otherwise uniform consortia of microbial genomes. Experimental methods of metatranscriptomics are not as developed as shotgun metagenomics, and similar to analyzing the metagenome, complications in bioinformatics often make it difficult to sort out true biological signals from background noise. Reference databases of culture-independent metagenomes and whole-genome sequenced isolates are also in the progress of enrichment with curation being standardized (Shakya et al., 2019).

#### Metaproteomics

Metaproteomics is large-scale identification and quantification of proteins from microbial communities which may provide insight into the phenotypes of microorganisms on the molecular level within a given system (Hinzke et al., 2019; Kleiner, 2019). These methods have recently enabled the quantification of per-species biomass to determine community structure, suggesting feasibility to determine differences in microbiomes between groups based on the detection of microbial proteins found systemically in the host. Building a reference database based on metagenomic sequencing data is often a prerequisite for metaproteomics. A well-curated protein sequence database would also benefit the assignment of proteins to individual species or higher taxa and contribute to understanding the functional roles and potential interactions of individual members in the community. Standardized metaproteomic data can be used to analyze community structure on the basis of biomass instead of gene/genome copy counts (metagenomics) (Kleiner, 2019). Unfortunately, a major technical factor that limits metaproteomics is the throughput for analysis, as the number of mass spectra that can be acquired by a given instrument is limited by time.

#### Metametabolomics

Aside from focusing on identifying microbial proteins of interest, understanding host-microbial metabolism can unveil insights into the interactions between the host and the microbiota, as well as to explore microbe-derived therapeutic products. Metabolic profiling of plasma was used to identify biomarkers to serve as predictors for acute anterior uveitis (AAU) progression and treatment response (Guo et al., 2014). Plasma metabolic biomarkers and metabolic pathways were compared between AAU and healthy subjects using ultra-performance liquid chromatography-mass spectrometry, and differentially abundant metabolic biomarkers and pathways were identified between AAU patients and healthy controls (Guo et al., 2014). A similar approach was used to identify differences in complement and coagulation cascades in the plasma of EAU rats, linking uveitis pathogenesis to complement activation (Guo et al., 2017).

Metabolomic studies can also be applied to the interrogation of intestinal microbiota (metametabolomics) (Tang, 2011; Noecker et al., 2019). Metametabolomics is the global study of small molecules or metabolites in a particular physiological state of a community (O’Malley, 2013), such as a sample of fecal content. For example, the metabolic phenotype of fecal samples collected from AAU patients was compared to healthy controls by analyzing the composition of microbiota through metagenomic and metabolomic methods (Huang et al., 2018). Although the results did not reveal compositional difference between healthy and AAU-associated gut microbiota, multivariate analysis showed that levels of several fecal metabolites were found to be increased in AAU patients (Huang et al., 2018).

#### Metaregulome

The entire suite of regulatory components in a cell is named the regulome, and its study has focused on processes by which a set of genes are regulated during development or under different physiological and pathological states (Kondro, 2004). Although the regulome is not a novel concept, recent advances in epigenome analysis methods (Chen et al., 2016) greatly enhanced our research capacity to examine the complex regulatory machinery of both the host and associated microbiota (metaregulome). The field of metaregulome has yet to see more studies with next generation approaches; however, there have been some efforts in exploring the metaregulatory elements from environmental samples using metagenomic data (Fernandez et al., 2014). Integrating regulomic data with the other omic matrices may greatly expand our understanding of cellular functions as well as cell-to-cell interactions.

The limitations of the “-omics” approaches, with the possible exception of metagenomics, are the high cost per sample and the requirement of high-level bioinformatics expertise and computational power to be able to process “big data”. Concerning uveitis, so far metagenomic and metabolomic profiles on patients have been studied (Guo et al., 2014; Huang et al., 2018), but we are not aware of literature on metatranscriptmic, metaproteomic or metaregulome aspects, which are at this time less well developed. As these technologies improve and mature, processing and analysis of these “big data” approaches should become more affordable and more attainable.

### Integration of Host and Microbial Measurements – A Multi-Omics Approach

**FIGURE 2:**
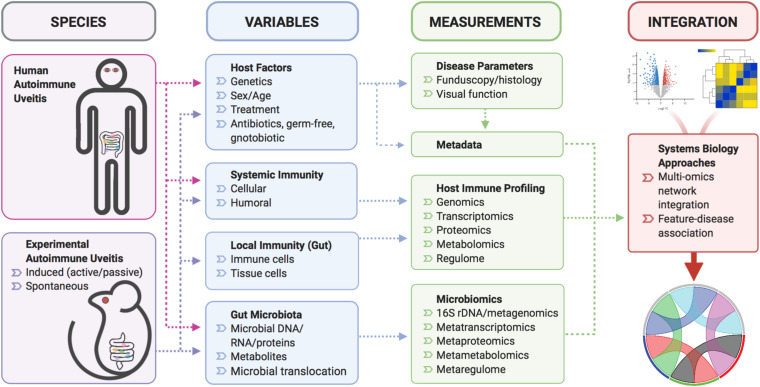
A schematic of multi-omic, systems biology approach for uveitis studies. Various mouse models of autoimmune uveitis or human uveitis patients will be sourced for blood and fecal samples. At the time of biological sample collection, variables that influence systemic/local immunity and the gut microbiota can be analyzed to yield data in the form of disease severity respective to host immune and microbiome status. These data could then be subjected to integrative multi-omic network analyses to provide meaningful relationships among various data types.

With the popularization of multi-omics technology, increasing numbers of studies incorporated metagenomic, metatranscriptomic, metaproteomic or metametabolomic analyses, the integration of which has led to unprecedented insights into mechanisms of disease-microbiome association (Figure 2). In a recent study exemplifying such multi-omic approach (as part of the Integrative Human Microbiome Project), researchers surveyed over a hundred human subjects of either a “healthy” or “inflammatory bowel disease (IBD)” status, and found that intestinal metagenomic, metatranscriptomic, and metabolomic profiles were disrupted during IBD activity (Lloyd-Price et al., 2019). Novel findings from the multi-omic integration are a collection of host-microbe molecular interactions that may underpin IBD activity, generated by a massive cross-measurement association network (Lloyd-Price et al., 2019). Multi-omic integrative analyses also revealed divergent mechanisms of microbiome perturbation on immunological response to vaccine administration (Krzywinski et al., 2009; Li et al., 2017; Hagan et al., 2019). In the study of flu vaccination in healthy subjects, broad-spectrum antibiotic treatment was shown to have a striking effect on the plasma metabolome, with microbiome-associated disturbances in bile acid metabolism highly correlated with elevated cellular transcriptional signatures of inflammation (Hagan et al., 2019). Although this study involved a small cohort of human volunteers, the insights gained from these limited samples can serve as a foundation upon which to build for future follow-up studies. Thus, a multi-omic approach has the potential to greatly accelerate biomarker discovery and therapeutics development, as well as to deepen our understanding of disease mechanisms, in studies of autoimmunity (Figure 2). The potential limitation can be accessibility/availability of patient samples for functional validation of proposed mechanisms gleaned from multi-omic approaches.

## Conclusion and Implications for Future Studies

Host-associated microbiota represent a dynamic ecosystem of commensal/pathogenic microbes, which are influenced by the environment and in turn affect our health. Experiments in laboratory-reared “clean” mice may not faithfully represent the human condition, as they cannot recapitulate the heterogeneity in genetic make-up and microbiota status in humans. Animal models such as “rewilded” and “wildling” systems, or possibly models with a “humanized” immune system, may help bridge this gap in some cases. In addition, it may be worth performing a thorough analysis of human patient samples to identify biomarkers of interest, prior to interrogating related pathways in animal models to determine disease mechanism.

Traditional approaches, including 16S metagenomic sequencing and immunological characterization, can be further enhanced by incorporating metabolic data from plasma or serum. Similar to how databases are being standardized, there is an urgent need to develop methods to analyze and integrate data from various sources in a systematic manner. Potentially, open-source analytical pipelines and containerized bioinformatics serve as a great opportunity to bridge the gap between big data and meaningful output of results. If implemented effectively, a systems biology approach and the integration of big data may yield new and exciting hypotheses to be tested.

## Author Contributions

RS and AZ made the outline and wrote the manuscript. RH and RRC supervised, edited, and finalized the manuscript. All authors contributed to the article and approved the submitted version.

## Conflict of Interest

The authors declare that the research was conducted in the absence of any commercial or financial relationships that could be construed as a potential conflict of interest.

## Footnotes

1 http://www.iedb.org/
